# A Streamlined DNA Tool for Global Identification of Heavily Exploited Coastal Shark Species (Genus *Rhizoprionodon*)

**DOI:** 10.1371/journal.pone.0034797

**Published:** 2012-04-09

**Authors:** Danillo Pinhal, Mahmood S. Shivji, Pedro G. Nachtigall, Demian D. Chapman, Cesar Martins

**Affiliations:** 1 Departament of Genetics, UNESP - São Paulo State University, Botucatu, São Paulo, Brazil; 2 Save Our Seas Shark Center and Guy Harvey Research Institute, Oceanographic Center, Nova Southeastern University, Dania Beach, Florida, United States of America; 3 School of Marine and Atmospheric Science & Institute for Ocean Conservation Science, Stony Brook University, Stony Brook, New York, United States of America; 4 Department of Morphology, UNESP - São Paulo State University, Botucatu, São Paulo, Brazil; American Museum of Natural History, United States of America

## Abstract

Obtaining accurate species-specific landings data is an essential step toward achieving sustainable shark fisheries. Globally distributed sharpnose sharks (genus *Rhizoprionodon*) exhibit life-history characteristics (rapid growth, early maturity, annual reproduction) that suggests that they could be fished in a sustainable manner assuming an investment in monitoring, assessment and careful management. However, obtaining species-specific landings data for sharpnose sharks is problematic because they are morphologically very similar to one another. Moreover, sharpnose sharks may also be confused with other small sharks (either small species or juveniles of large species) once they are processed (i.e., the head and fins are removed). Here we present a highly streamlined molecular genetics approach based on seven species-specific PCR primers in a multiplex format that can simultaneously discriminate body parts from the seven described sharpnose shark species commonly occurring in coastal fisheries worldwide. The species-specific primers are based on nucleotide sequence differences among species in the nuclear ribosomal internal transcribed spacer 2 locus (ITS2). This approach also distinguishes sharpnose sharks from a wide range of other sharks (52 species) and can therefore assist in the regulation of coastal shark fisheries around the world.

## Introduction

Shark fisheries have intensified worldwide in response to increasing demand for shark products (fins, meat). A major impediment managing these shark fisheries is the lack of species-specific catch data. Most shark landings are reported as an amalgam of species, in which products are sorted by broad taxonomic groups. This problem is compounded by the fact that sharks are not usually sold as whole animals, but as carcasses or processed in the form of fillets, making it difficult to identify species of origin.

**Figure 1:**
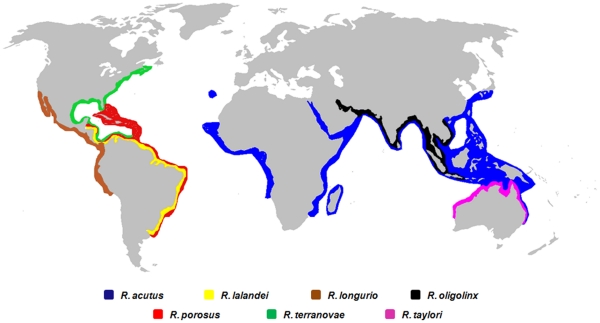
Global oceanic distribution of sharpnose sharks. Species geographical ranges were based on data available at Florida Museum of Natural History (http://www.flmnh.ufl.edu/fish/) and Fishbase (http://www.fishbase.org/) websites. World map in raw version was taken from wikipedia.

The genus *Rhizoprionodon* is represented worldwide by seven species of small sharks [1] characterized by a relatively pointed snout, earning them the common name “sharpnose sharks”: *Rhizoprionodon porosus* (Caribbean sharpnose shark), *R. lalandei* (Brazilian sharpnose shark), *R. terranovae* (Atlantic sharpnose shark*)*, *R. oligolinx* (Grey sharpnose shark), *R. taylori* (Australian sharpnose shark), *R. acutus* (Milk shark) and *R. longurio* (Pacific sharpnose shark). All these sharks are range-restricted and inhabit tropical and subtropical inshore waters in either the Atlantic, Indian or Pacific oceans [1], [2] (Figure 1). Sharpnose sharks exhibit a conserved external morphology that makes them quite difficult to identify, even as whole animals.

Sharpnose sharks are landed in large quantities by artisanal and commercial fisheries in many locations worldwide [3], [4]. They exhibit life history characteristics such as rapid growth, early maturation and an annual reproduction cycle that make them among the most productive shark species and therefore presumably less susceptible to overexploitation than many of the larger sharks. This suggests that sharpnose sharks may be sustainable in modest fisheries, assuming sound management including the monitoring of species landings and population trends. The International Union for the Conservation of Nature (IUCN) red list of threatened species database lists *R. lalandei* and *R. longurio* as “Data deficient” for assessment [4], [5], but the remaining species are categorized as “least concern or low risk”. In all cases, however, these designations are based on basic life history information rather than landings or population trend data, with the exception of *R. porosus*, which is considered to exhibit a “stable” population trend. There is some evidence for decline in some of the Atlantic species due to fisheries overexploitation [6]. Despite broadly similar life histories, different sharpnose species still may respond differently to local fishing pressure, making it important to monitor, assess and manage them on a species rather than on a group-specific basis [7]–[9]. This is problematic given the difficulties in identifying sharpnose sharks and their products.

Molecular methods have previously been used to quantify global shark fin landings, detect or reveal captures of threatened shark species and trace the geographic origin of shark parts in trade [10]–[12]. An economical and streamlined genetic species identification method developed for sharks involves a multiplex PCR format [13]–[17]. This approach uses multiple, species-specific primers in a single-reaction tube to produce diagnostic amplicons from the nuclear ribosomal DNA Internal Transcribed Spacer 2 (ITS2) locus. The species-specific primers are based on consistent nucleotide sequence differences among species in their ITS2 locus. Unlike RFLP or phylogenetic analysis used in many species identification studies, this approach requires only PCR without additional enzymatic processing or sequencing of the amplified products to derive a species diagnosis.

The objective of the present study was to develop a multiplex PCR assay to simultaneously identify all seven heavily exploited species of sharpnose sharks. The main goal was the obtainment of a complete set of species-specific primers for monitoring the global catch and trade on sharpnose species. This methodology was further validated through screening of market-derived samples in two independent case studies.

## Methods

### Shark Sampling

One hundred sixty six sharpnose shark fin and muscle tissue samples used for ITS2 DNA sequencing and species-specific primer testing (hereafter referred to as “reference samples”) were collected from fresh or frozen specimens caught by local fishermen and commercial boats in coastal areas of the Atlantic, Indian and Pacific Oceans (Table 1). These reference samples for all seven *Rhizoprionodon* were specifically collected from southern and northern Brazil (*R. lalandei* and *R. porosus*), Gulf of Mexico, southern USA (*R. terranovae*), Oman and Kuwait coast, (*R. oligolinx*), northern and western Australia (*R. acutus* and *R. taylori*, respectively) and western Mexico (*R. longurio*). Putative species-specific sharpnose primers were also tested against reference samples from 52 additional shark species (Table S1). These samples compose a shark inventory managed by Dr. Shivji at the Conservation Genetics Lab, Oceanografic Center, Florida. All sharks used in this study were identified by experienced shark researchers. Samples were preserved in 95% ethanol and kept at -4°C for long-term storage.

**Table 1:** Inventory of sharpnose sharks showing the number of individuals investigated by species and their geographic ocean basin origins.

Species	Geographic origin (n)
***Rhizoprionodon lalandei*** (Brazilian sharpnose)	Atlantic (30)
***Rhizoprionodon porosus*** (Caribbean sharpnose)	Atlantic (32)
***Rhizoprionodon terranovae*** (Atlantic sharpnose)	Atlantic (40)
***Rhizoprionodon longurio*** (Pacific sharpnose)	Pacífic (21)
***Rhizoprionodon acutus*** (milk shark)	Pacífic (17)
***Rhizoprionodon taylori*** (Australian sharpnose)	Pacific (12)
***Rhizoprionodon oligolinx*** (grey sharpnose)	Índian (14)

### DNA Extraction, PCR and Sequencing

Total genomic DNA was isolated from muscle or fin clips using the DNeasy Tissue Kit (Qiagen Inc.) following the manufacturer instructions. The whole ITS2 region was amplified using the universal primers FISH5.8SF and FISH28SR [13]. PCRs contained 10–25 ng of extracted DNA, 12.5 pmol of each primer, 2.0 mM of MgCl_2_, 1x PCR buffer, 200 µM dNTP’s and 1 unit of Platinum Taq DNA polymerase (Invitrogen Inc.). The reactions were performed on an iCycler (BioRad) thermal cycler. Cycling profiles consisted of initial heating at 94°C for 3 min, followed by 35 cycles at 94°C for 1 min, 55°C for 1 min, 72°C for 2 min and a 5 min extension at 72°C. Amplified segments were visualized on a 1% agarose gel stained with *GelRed* (Uniscience), under UV light. All products were purified with the QIAquick PCR purification kit using the manufacturer’s protocol (Qiagen Inc.).

Sequencing reactions were performed using the BigDye Terminator v3.1 kit (Applied Biosystems, Inc), following the manufacturer’s protocol. The cycling profile comprised an initial denaturation at 95°C for 5 min, followed by 35 cycles of denaturation at 95°C for 1 min, annealing at 50°C for 30 s and extension at 60°C for 1 min. Products were gel purified using the DyeEx 2.0 Spin Kits (Qiagen Inc) and sequencing was carried out on a Applied Biosystems 3130 DNA analyzer (Applied Biosystems, Inc.). The final ITS2 locus sequence size retrieved from each species is shown on Table 2.

**Table 2:** Size of ITS2 locus of sharpnose sharks excluding 5.8S and 28S rRNA gene flanking regions.

Species	ITS2 size (bp)
*R. taylori*	1282
*R. acutus*	1311
*R. oligolinx*	1326
*R. lalandei*	1355
*R. porosus*	1361
*R. terranovae*	1363
*R. longurio*	1365

### Primers Design and PCR Multiplex

**Figure 2:**
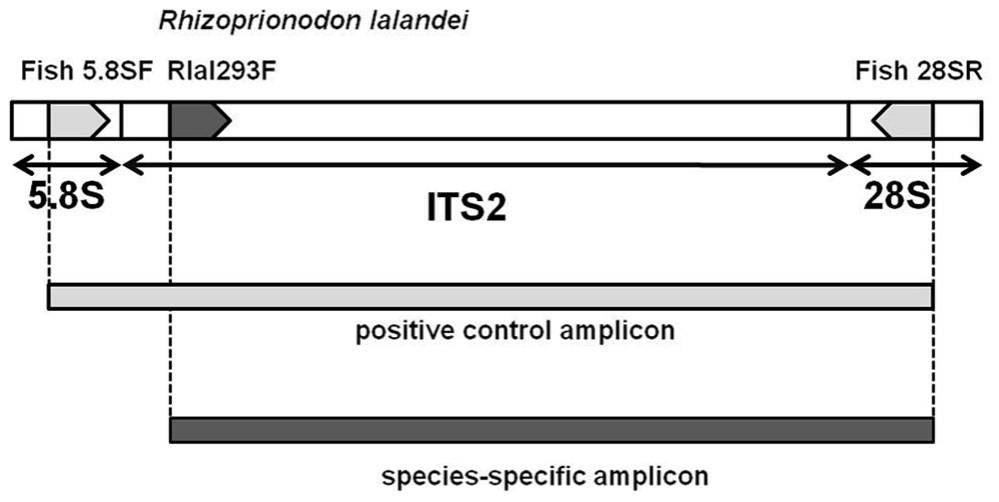
Triplex scheme of ITS2 species-diagnostic primers. Representation of the shark nuclear 5.8S and 28S ribosomal RNA genes and ITS2 locus showing relative annealing sites and orientation of the shark universal ITS2 primers (Fish 5.8SF and 28SR indicated by gray irregular pentagons). The Brazilian sharpnose (*R. lalandei*) Rlal293F primer is an example of a species-specific primer used in this study and is shown as a dark gray irregular pentagon. Also represented are the positive control and species-specific amplicons expected to be produced using this combination of three primers when tested against the target species, *R.lalandei*, DNA (Figure adapted from Shivji et al. 2002).

Complete ITS2 locus sequences acquired from all seven sharpnose sharks were aligned using GENEIOUS (Biomatters, Ltd) and the genetic distances among species calculated in MEGA 4 [18]. Then several putative species-specific primers (“SSPs”) were designed for each one of the seven species based on the nucleotide differences found between the target sequence and the other non-target sharpnose species using the programs Primer3plus [19] and OligCalc [20]. Putative SSPs were initially tested individually against each *Rhizoprionodon* species (sample sizes in Table 1) in a mixture of three primers in a PCR multiplex (triplex) format that also included the forward and reverse shark universal ITS2 primers [13]. In principal, two amplification products are expected from the target species with this triplex PCR: a species characteristic-sized PCR amplicon generated by the forward SSP in conjunction with the reverse ITS2 universal primer, and a positive control amplicon generated by the two ITS2 universal primers (Figure 2). In contrast, DNA from non-target species is expected to yield only the positive control amplicon owing to failure of the species-specific primer to anneal to non-target genomic DNA [13].

Triplex-PCR reactions were standardized after gradient temperature tests, resulting in the following optimized conditions: denaturation at 94°C for 3 min, followed by 35 cycles at 94°C for 1 min, 65°C for 1 min, 72°C for 2 min and a 5 min extension at 72°C. Amplifications were carried out in a MJ Research PTC 200 thermal cycler (MJ Research Inc.). The putative SSPs that consistently amplified the correct-sized fragment for their respective target species but not any other *Rhizoprionodon* congeners were then further tested for their species-specificity (same cycling conditions as above) against 52 additional non-target (i.e., non-*Rhizoprionodon*) shark species representing a wide range of evolutionarily diverse lineages and also known to occur in fisheries (Table S1). After these preliminary tests, one final SSP was selected for each of the seven target sharpnose species. SSPs were selected to ensure that each one produced an amplicon of a diagnostic size when used in the subsequent larger multiplex PCR format.

**Figure 3:**
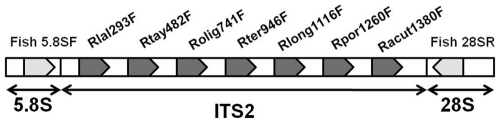
Nonaplex scheme of ITS2 species-diagnostic primers. Representation of the shark nuclear 5.8S and 28S ribosomal RNA genes and ITS2 locus showing relative annealing sites and orientation of primers used in the nonaplex-PCR assay. Shark universal primers (Fish 5.8SF and Fish 28SR) are shown as gray irregular pentagons, while the seven sharpnose species-specific primers are shown by dark gray irregular pentagons. Rlal: *Rhizoprionodon lalandei*; Rtay: *R. taylori*; Rolig: *R. oligolinx*; Rter: *R. terranovae*; Rlong: *R. longurio*; Rpor: *R. porosus*; Racut: *R. acutus*.

### Developing a Multiplex Assay to Distinguish Seven *Rhizoprionodon* Species Simultaneously

To establish a further streamlined assay for identification of all *Rhizoprionodon* species in international trade, we tested a nine-primer (nonaplex) PCR assay for its diagnostic performance. This nonaplex format consisted of the seven sharpnose SSPs plus the two shark universal primers (Figure 3). This assay was expected to yield a diagnostic-sized, species-specific amplicon plus an ITS2 positive control amplicon from each of the seven sharpnose species. In contrast, only a single positive control amplicon representing the whole ITS2 locus was predicted using the DNA from any non-target shark species. Owing to size variability of the ITS2 locus in shark species [16] [18], the positive control amplicon from non-target sharks was expected to range from ∼860 bp to 1500 bp. We tested the nonaplex assay against all reference samples under the same cycling conditions used for the triplex assay.

**Table 3:** Genetic distances within and between sharpnose sharks calculated as pairwise Tamura-Nei for the nuclear ITS2 locus.

ITS2	*R. acutus*	*R. porosus*	*R. taylori*	*R. terranovae*	*R. lalandei*	*R. longurio*	*R. oligolinx*
*R. acutus*	**N/C**						
*R. porosus*	0.205	**0.001**					
*R. taylori*	0.272	0.214	**N/C**				
*R. terranovae*	0.208	0.003	0.216	**0.000**			
*R. lalandei*	0.205	0.015	0.219	0.016	**N/C**		
*R. longurio*	0.206	0.012	0.221	0.015	0.018	**0.001**	
*R. oligolinx*	0.226	0.059	0.235	0.061	0.063	0.061	**N/C**

N/C: intra-specific genetic distances not calculated since only one animal sequenced.

### Market-derived Shark Product Survey

All the seven sharpnose species-specific primers developed in this study along with SSPs available for some non-sharpnose shark species [14]–[16] were utilized for screening market derived samples. First, we used these primers to identify 69 pieces of shark meat from unknown specimens sourced at the Canto do Mangue and Alecrim fish markets in Natal, RN, northeast coast of Brazil. In the second study, these primers were applied to check the identity of 21 shark carcasses acquired from fisherman in two fish markets in the Macaé harbour, RJ, southeast coast of Brazil. After identifying them with SSPs, market-derived samples were sequenced and compared to reference sharpnose sequences. The sequencing protocol used was performed as previously described.

**Table 4:** Species-specific primers designed along with their sequences and expected amplicon sizes for each species.

Species-specific primers	Primer sequences	PCR product sizes (bp)
Racut-555 ITS2	5′ TTAACGTTCTGTGCGTGTCGAGT3′	230 pb
Rpor-1260 ITS2	5′GCGAGGCACACCTCGGCAC3′	420 pb
Rlong-1116 ITS2	5′GACTTGCTCTGTCCTTGAGCCC3′	560 pb
Rter-946 ITS2	5′TGTGAATAGGGGCAGCCGACA3′	720 pb
Rolig-741 ITS2	5′TACCGGGAGAGCTCGGAAAACGT3′	850 pb
Rtay-482 ITS2	5′AACGGTTCGGGTGCTCCGGCA3′	1150 pb
Rlal-293 ITS2	5′GGCACGTAGGCACCGCCCGCTAT3′	1300 pb

Rlal: *Rhizoprionodon lalandei*; Rtay: *R. taylori*; Rolig: *R. oligolinx*; Rter: *R. terranovae*; Rlong: *R. longurio*; Rpor: *R. porosus*; Racut: *R. acutus*.

## Results

### Evaluating the Species-specific Primers in the Multiplex Assays

All target and non-target shark species evaluated in the triplex and nonaplex PCR assays and their geographic source and sample sizes are listed in Tables 1 and S1. The ITS2 locus for sharpnose sharks ranged from 1282 to 1365 bp (Table 2; GenBank accession numbers: JN008711-JN008720). Intra and interspecific genetic distances among sharpnose species are shown in Table 3. The sequences and the size of the amplicon produced for each sharpnose shark species-specific primer are listed in Table 4.

**Figure 4:**
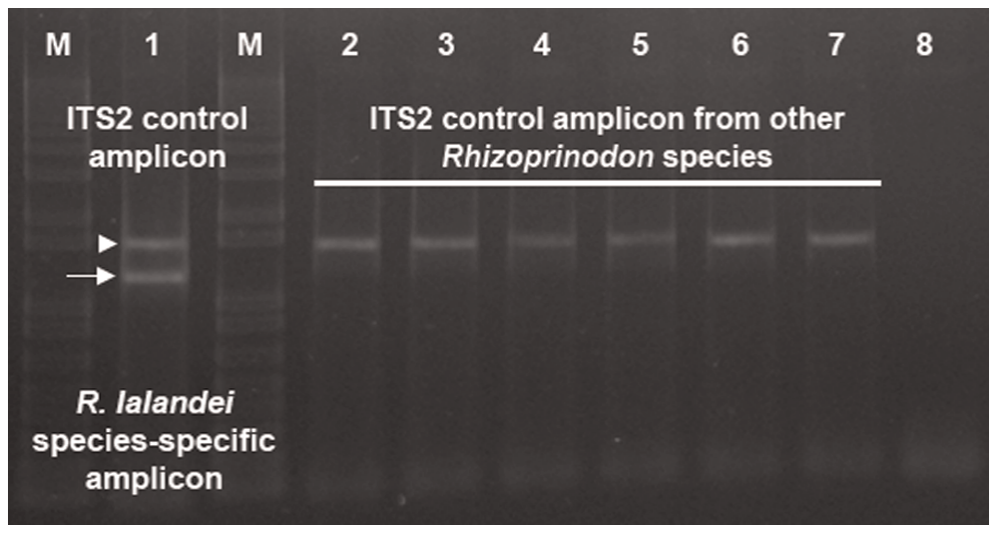
Triplex primer amplication gel profile. Results of amplification reactions utilizing the triplex primer combination of two shark ITS2 universal primers and the *R. lalandei* species-specific primer Rlal293F (Lanes 1 to 7). Lane 1 shows the target species-specific (arrow) and positive control (arrowhead) amplicons. Lanes 2–7 show amplification products from non-target *Rhizoprionodon* species tested for Rlal293F primer cross-amplification: 2, *R. porosus*; 3, *R. terranovae*; 4, *R. acutus*; 5, *R. longurio*; 6, *R. oligolinx*; 7, *R. taylori*; 8, Negative control (no shark DNA in the PCR). Lanes labeled M contain the molecular size-standard 1 kb plus.

**Figure 5:**
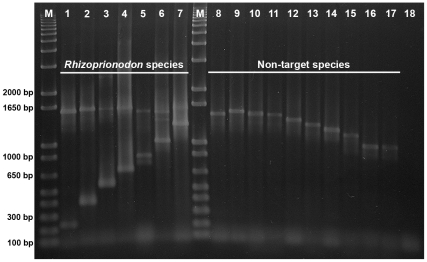
Nonaplex primer amplification gel profile. Results of amplification reactions utilizing the nine-primer nonaplex: two shark universal primers and all the seven species-specific primers. Lanes 1–7 show nonaplex-PCR amplification products of target *Rhizoprionodon* samples: 1- *R. acutus*; 2- *R. porosus*; 3- *R. longurio*; 4- *R. terranovae*; 5- *R. oligolinx*; 6- *R. taylori*; 7- *R. lalandei*. Lanes 8–17 show nonaplex-PCR amplification products from non-target species: 8- *Galeocerdo cuvier*; 9- *Negaprion acutidens*; 10- *Carcharhinus porosus*; 11- *Prionace glauca*; 12- *Isurus paucus*; 13- *Alopias superciliosus*; 14- *Squalus acantias*; 15- *Nebrius ferrugineus*; 16- *Squatina californica*; 17- *Hexanchus griseus*. Lines 8–11, Carcharhiniformes; Line 12, Orectolobiformes; Line 13, Squaliformes; Lines 14–15, Lamniformes; Line 16, Squatiformes; Line 17, Hexanchiformes; Line 18 is the negative control. Lanes labeled “M” contain the molecular size-standard 1kb plus. Faint non-specific bands likely correspond to pseudogenes or uncommon variant copies of ribosomal genes rarely amplified by universal and species-specific primers.

The seven final, sharpnose shark SSPs exhibited complete species-specificity in individual triplex PCR assays (example shown in Figure 4) on the sample sizes we were able to obtain for each species. In the nonaplex PCR format, all seven SSPs maintained their species-specificity in relation to target and non-target species (Figure 5). However, the co-amplification of the positive control ITS2 amplicon in both triplex and nonaplex assays from target species was inconsistent (variably faint), as previously reported in similar assays for other shark species [14], [16]. Importantly, in non-target species testing only the positive control amplicons (i.e., no false positive identifications) were produced in all cases (e.g., Figures 4 and 5).

### Market Screening

Of the 69 shark meat samples tested, 35 samples were genetically identified by SSPs as originating from two sharpnose species (34 *R. porosus* and one *R. lalandei*). Other 34 meat samples comprised the following: 12 blue sharks (*Prionace glauca*), 11 shortfin mako sharks (*Isurus oxyrhinchus*), two scalloped hammerhead sharks (*Sphyrna lewini*), and a single sample each from silky (*Carcharhinus falciformis*), dusky (*C. obscurus*) and white shark (*Carcharodon carcharias*). The remaining six samples were from unidentified species likely not corresponding to the set of species-specific primers utilized. Of the 21 carcasses tested, seven were Brazilian sharpnoses, three were Caribbean sharpnoses, five were scalloped hammerheads and six were smooth hammerheads (*Sphyrna zygaena*). Posterior sequencing of all market-derived shark samples undoubtedly confirmed their IDs provided by SSPs (data available upon request to the authors).

## Discussion

All sharpnose shark SSPs developed were observed to reliably amplify only their respective target species and none of the 52 non-target species tested in both triplex and nonaplex PCR formats, confirming their potential species-diagnostic utility. The large dataset evaluated (166 reference samples) and the large interspecific genetic divergence found in the ITS2 locus among all sharpnoses (Table 3) indicates that the possibility of false positive (i.e., cross species amplification) or false negative (i.e., failure to identify a sharpnose species) is remote. Indeed, the ITS2 locus has been used for species identification owing to its consistent low intraspecific polymorphism and high interspecific variability even among closely related congeneric species [13], [15]. Furthermore, the ITS2 locus is part of the tandemly organized 45S ribosomal DNA repeats [21], which means an abundance of target sites for primer annealing and an improved amplification by PCR. Together these features underscore the ability of the methodology proposed for genetic identification of sharpnose sharks. The identifications of the unknown market-sourced samples by PCR were all later confirmed by sequencing the ITS2 locus, further attesting the reliability of the primer assay.

During standardization of the nine-primer multiplex methodology, the positive control amplicon was faintly amplified in some of the target sharpnose sharks as previously reported for other species [15], [16]. Nevertheless it was always present for the seven target species after fine tune adjustments in the PCR (e.g., increase of MgCl2 concentration and longer extension time). The lower efficiency of the universal primers relative to the SSPs is probably caused by the large-sized positive control amplicon generated by the two ITS2 universal primers in *Rhizoprionodon*. In previous studies using the PCR-multiplex approach for species identification, the positive control was included only to prevent the complete absence of any amplification (e.g., from PCR reaction failure) from being interpreted as the absence of a particular species (i.e., a false-negative result) [13], [14]. However in instances of this study, as to distinguish some *Rhizoprionodon* species with larger species-diagnostic amplicons from smaller positive control ITS2 amplicons of equivalent molecular weight (e.g., hammerheads and sixgills), the presence of an accompanying positive control amplicon was essential, allowing undoubted species diagnosis.

Our assay provides a relatively inexpensive and straightforward procedure to (1) identify products as originating from a sharpnose shark and (2) discriminate amongst sharpnose species. The assay is ready to be applied in the acquisition of catch and trade data for these species anywhere in the world. The advantage of one-tube reaction is that it enables shorter PCR cycles and good results with minor amounts of template DNA, a feature particularly functional in forensic applications [15], [22], [23]. The assay also improves on existing methods to identify sharpnose sharks (cytochrome oxidase I barcode sequences) [24], because it is far more economically and less time consuming to do PCR than sequencing. Furthermore, most sharpnose sharks are primarily exploited in developing nations where the costs of DNA sequencing are currently likely be prohibitive. Previous work provided a COI-based SSP approach for the identification of *R. lalandei* and *R. porosus* [25]. Our assay improves on this by allowing for the identification of the remaining Atlantic species: *R. terranovae*. Combinations of these three species have an overlapping range in many locations in the Atlantic. The ability to identify all three species at once ensures that this one assay can be used throughout the Atlantic. Routine application of this assay to monitor fisheries may result in a clearer understanding of the distribution and degree of overlap between these similar-looking species.

Many shark populations are fished at or above a level that is sustainable and there is an urgent monitor and regulate shark fisheries. Although more data is needed to determine whether this is the case or not for several of the sharpnose species, their relatively high productivity suggests that this group of sharks could sustain a level of properly managed fishing pressure. Our assay provides an expedient and inexpensive procedure that could be used to monitor the catch of sharpnose sharks on a species-specific basis. We envision it will be most useful in developing nations that simultaneously fish several sharpnose species at once, especially in the Western Atlantic (Caribbean, South America). The case studies presented here clearly addressed this issue and the genetic identification provided by our multiplex PCR approach demonstrates its utility. It could also be used in conjunction with other DNA identification methods to rapidly separate sharpnose products from similar products from juveniles of larger species, to better understand how inshore fisheries affect recruitment of larger, more vulnerable shark species.

## Supporting Information

Table S1 Inventory of non-target shark species tested with the sharpnose shark species-specific primers in triplex and nonaplex PCR assays. Geographic ocean basin origins of the shark test species are shown, with (n) representing the number of individuals of each species tested from each geographic region.
(DOC)
