# Supplementary material for: A Streamlined DNA Tool for Global Identification of Heavily Exploited Coastal Shark Species (Genus Rhizoprionodon)
Source: PLoS One. 2012 Apr 9;7(4):e34797. doi: 10.1371/journal.pone.0034797 (PMC3322161; doi:10.1371/journal.pone.0034797)
Supplement: Table S1 — Inventory of non-target shark species tested with the sharpnose shark species-specific primers in triplex and nonaplex PCR assays. Geographic ocean basin origins of the shark test species are shown, with (n) representing the number of individuals of each species tested from each geographic region. (DOC) [file pone.0034797.s001.doc]

**Table S1.** Inventory of non-target shark species tested with the sharpnose shark species-specific primers in triplex and nonaplex PCR assays. Geographic ocean basin origins of the shark test species are shown, with (n) representing the number of individuals of each species tested from each geographic region.

| **Species** | **Geographic origin (n)** |
| --- | --- |
| **ORDER CARCHARHINIFORMES** |  |
| *Sphyrna mokarran* (great hammerhead) | Atlantic (3); Pacific (3) |
| *Sphyrna lewini* (scalloped hammerhead) | Atlantic (5); Pacific (3) |
| *Sphyrna zygaena* (smooth hammerhead) | Atlantic (5); Pacific (3) |
| *Sphyrna tiburo* (bonnethead) | Atlantic (5) |
| *Sphyrna tudes* (golden hammerhead) | Atlantic (5) |
| *Eusphyra blochii* (winghead) | Pacific (3) |
| *Isogomphodon oxyrinchus* (Daggernose shark) | Atlantic (3) |
| *Loxodon macrorhinus* (sliteye) | Atlantic (6) |
| *Galeocerdo cuvier* (tiger) | Atlantic (5); Pacific (3) |
| *Triaenodon obesus* (whitetip reef) | Pacific (5) |
| *Prionace glauca* (blue shark) | Atlantic (7); Pacific (4) |
| *Carcharhinus altimus* (bignose) | Atlantic (3); Pacific (2) |
| *Carcharhinus longimanus* (oceanic whitetip) | Atlantic (2); Pacific (3) |
| *Carcharhinus signatus* (night) | Atlantic (5) |
| *Carcharhinus plumbeus* (sandbar) | Atlantic (2); Pacific (3) |
| *Carcharhinus obscurus* (dusky) | Atlantic (2); Pacific (3) |
| *Carcharhinus limbatus* (blacktip) | Atlantic (3); Pacific (3) |
| *Carcharhinus falciformis* (silky) | Atlantic (2); Pacific (2); Indian (1) |
| *Carcharhinus porosus* (smalltail) | Atlantic (5) |
| *Carcharhinus galapagensis* (galápagos) | Pacific (5) |
| *Carcharhinus leucas* (bull) | Atlantic (8); Pacific (3) |
| *Carcharhinus brevipinna* (spinner) | Atlantic (2); Pacific (5) |
| *Carcharhinus isodon* (finetooth) | Atlantic (5) |
| *Carcharhinus acronotus* (blacknose) | Atlantic (5) |
| *Carcharhinus perezi* (Caribbean reef) | Atlantic (5) |
| *Carcharhinus amboinensis* (pigeye) | Pacific (2); Indian (3) |
| *Carcharhinus brachyurus* (bronze whaler) | Atlantic (2); Pacific (3) |
| *Carcharhinus tilstoni* (Australian blacktip) | Pacific (5) |
| *Carcharhinus sorrah* (spot-tail) | Índian (2); Pacific (4) |
| *Carcharhinus amblyrhynchos* (gray reef) | Pacific (5) |
| *Negaprion brevirostris* (lemon) | Atlantic (5) |
| *Negaprion acutidens* (sicklefin lemon) | Pacific (5) |
| *Mustelus norrisi* (smoothhound) | Atlantic (2) |
| *Mustelus canis* (smooth dogfish) | Atlantic (6) |
| *Mustelus californicus* (grey smoothhound) | Pacific (1) |
| *Triakis semifasciata* (leopard) | Pacific (1) |
| *Scyliorhinus sp* (catshark) | Atlantic (6) |
| *Apristurus profundorum* (smalleye catshark) | Atlantic (1) |
| **ORDER LAMNIFORMES** |  |
| *Isurus oxyrinchus* (shortfin mako) | Atlantic (5); Pacific (3) |
| *Isurus paucus* (longfin mako) | Atlantic (2); Pacific (2) |
| *Lamna nasus* (porbeagle) | Atlantic (2); Pacific (3) |
| *Carcharodon carcharias* (white shark) | Atlantic (2); Indian (3); Pacific (3) |
| *Alopias vulpinus* (thresher) | Atlantic (1) |
| *Alopias superciliosus* (bigeye thresher) | Atlantic (5) |
| *Carcharias taurus* (sandtiger) | Atlantic (2); Indian(2); Pacific (2) |
| **ORDER ORECTOLOBIFORMES** |  |
| *Ginglymostoma cirratum* (nurse) | Atlantic (10) |
| *Nebrius ferrugineus* (tawny nurse) | Pacific (2) |
| **ORDER SQUALIFORMES** |  |
| *Squalus acanthias* (spiny dogfish) | Atlantic (4) |
| *Centrophorus squamosus* (leafscale gulper) | Atlantic (1) |
| **ORDER SQUATINIFORMES** |  |
| *Squatina californica* (Pacific angel) | Pacific (2) |
| **ORDER HEXANCHIFORMES** |  |
| *Hexanchus griseus* (sixgill) | Pacific (3) |
| *Heptranchias perlo* (sharpnose sevengill) | Atlantic (1) |
